# Citalopram & escitalopram: Mechanisms of cardiotoxicity, toxicology predisposition and risks of use in geriatric & hemodialysis populations

**DOI:** 10.21542/gcsp.2024.34

**Published:** 2024-08-01

**Authors:** Hadi Farhat, Yehya Tlaiss, Lea Nassif, Sai Dheeraj Gutlapalli, Razan Abdulaal

**Affiliations:** 1 Internal Medicine, University of Balamand, Beirut, Lebanon; 2 Internal Medicine, Richmond University Medical Center Mount Sinai, Staten Island, New York, USA; 3 Cardiology, University of Balamand, Beirut, Lebanon

## Abstract

The selective serotonin reuptake inhibitors (SSRIs) citalopram and escitalopram are extensively prescribed for various psychopathies. Despite their reputation for safety compared to older antidepressants, concerns have arisen regarding their cardiotoxic potential, notably in prolonging the QTc interval. In this comprehensive review, we investigate the intricate mechanisms of cardiotoxicity induction by citalopram/escitalopram, with a special focus on their interactions with ion channels like Kv11.1, Nav1.5, and Cav1.2 which may contribute to QTc-prolongation, increasing the risk of life-threatening arrhythmias such as Torsades de Pointes (TdP). Moreover, we explore the predisposing factors to their associated cardiotoxicity along with an investigation of the QRS/QTc ratio as a potential biomarker for identifying patients at risk of ventricular arrhythmias, taking into consideration the impact of genetic variations and drug interactions, especially those involving the liver CYP2C19 metabolism. Our review extends to the geriatric population’s use of citalopram and escitalopram, emphasizing the significance of assessing a patient’s medical history and cumulative drug use to evaluate their susceptibility to cardiac adverse events. Finally, we scrutinize the compound relationship between QTc-prolongation, proton pump inhibitors (PPIs) and serum-to-dialysate potassium gradients in influencing the proarrhythmic potential of citalopram/escitalopram in hemodialysis patients.

## Introduction

Citalopram (sold under the tradename Celexa^®^) belongs to the selective serotonin reuptake inhibitor (SSRI) class of drugs. Citalopram is a racemic mixture of the enantiomers R-citalopram and S-citalopram (aka escitalopram; sold under the tradename Lexapro^®^)^1,2^, with the latter having a higher safety profile with less adverse effects in terms of severity and frequency^1^.

Citalopram and escitalopram, are the recommended first-line treatment for multiple psychiatric disorders including depression and anxiety^3,4^, are generally thought to be safer than alternatives such as tricyclic antidepressants^4^. However, despite being efficacious and well-tolerated, their use has been linked to prolonged QTc-interval^1,2,4–7^, cardiotoxicity^1^, and even fatal cardiotoxic events^1,2^.

While central nervous system depression, seizures, serotonin syndrome, possible peripheral artery disease in diabetics and cardiac electrophysiological abnormalities could be side effects after a citalopram overdose, little data exists concerning seizures and cardiac toxicity with escitalopram^2,8^. Nevertheless, both citalopram and escitalopram can lead to cardiac conduction problems when administered at high doses^1^.

A Drug Safety Communication issued by the US Food and Drug Administration (FDA), in 2011^9^ and 2012^10^ recommended maximum doses of citalopram of 20 mg (no longer 40 mg) for over 60 year-olds due to risk of QT prolongation and development of the potentially fatal ventricular arrhythmia Torsades de Pointes (TdP)^9,10^.

This review includes an overview of the potential mode of induction of conduction problems, the predisposing factors for cardiotoxicity, the risk of QTc prolongation in geriatric setting, and the modifying effects of their use in the hemodialysis population.

## Methods

Our literature review drew upon pertinent data sourced exclusively from the PubMed database, a trusted and authoritative repository for scholarly research. We employed a refined search strategy, utilizing four key search terms: “Citalopram,” “Escitalopram,” “QTc prolongation,” “Torsades de Pointes,” and “Cardiac Toxicity.” This search was conducted following the comprehensive Medical Subject Heading (MeSH) strategy. To ensure the inclusion of the most up-to-date and relevant information, we diligently screened and incorporated recent articles available until August 2nd, 2023. All information referenced in our study originates from the PubMed database, reflecting its reliability and credibility as a primary data source.

## Risk of arrhythmias

### Citalopram & escitalopram induction of cardiotoxicity

**Figure 1.**
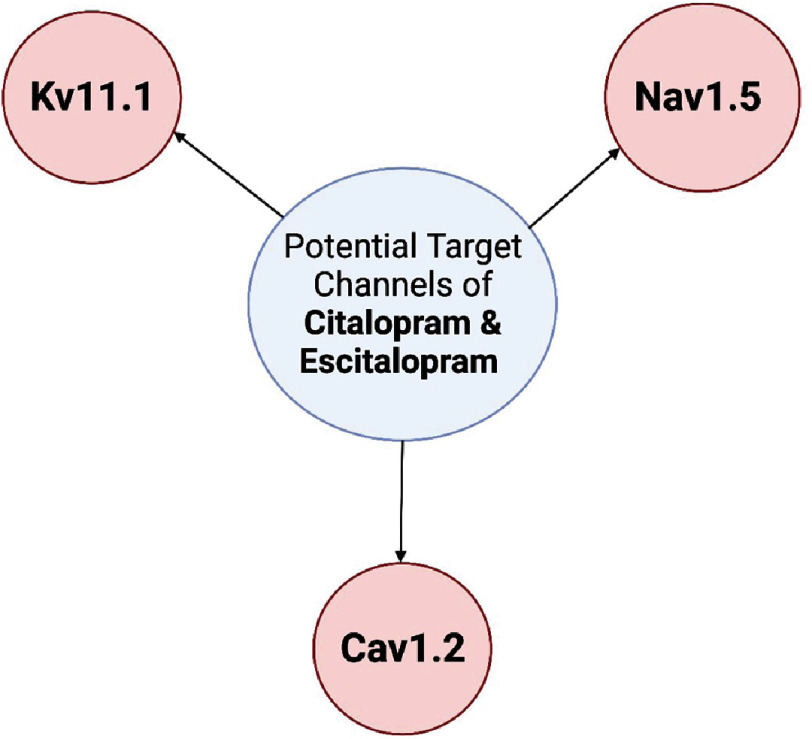
Voltage-gated target channels of citalopram/escitalopram.

One of the important developing disciplines in pharmacology is Safety Pharmacology which aims to assess the potential risks of pharmacotherapy. This becomes especially important when toxic effects of a certain drug start to affect vital organs. An anti-target is “an undesirable molecular target that plays an essential role in the proper functioning of cells”^7^. Downregulation of an anti-target can end up initiating disease or modifying disease progression^7^. Numerous heart diseases are due to disruption of normal ion channel functioning. Consequently, many studies have been done to review selected potential antitargets, including the voltage-gated channels like Kv11.1, Nav1.5 and Cav1.2, for cardiotoxicity (Figure 1).

The Kv11.1 channel, encoded by the human Ether-à-go-go-Related Gene (hERG), plays a fundamental role in cardiac repolarization^7^. Formally, QTc-prolongation has been linked to the blockade of the hERG K+ channel^1^, and since both citalopram and escitalopram can inhibit those channels too, hERG K+ channels could be key molecular targets for inducing prolongation of the QTc interval^1,7^.

Additionally, Nav1.5 voltage-gated sodium channel (VGSC) is main anti-target for drug cardiotoxicity. Nevertheless, gain-of-function and loss-of-function mutations of the Nav1.5 gene can induce QTc prolongation^1,7^.

Escitalopram has been suggested to be the cause of QRS complex widening due to change in functioning of sodium channels^1^. Research has been conducted to inspect whether citalopram or one of the constituents’ enantiomers escitalopram (S) or R-citalopram primarily affected Nav1.5 VGSC on cardiomyocytes. Results showed all enantiomers inhibited Nav1.5 VGSC peak current in a concentration-dependent manner, and that inhibition might be reversible. Differences in the inhibitory effects of citalopram, escitalopram and R-citalopram were noted at a concentration of 100 μM, but were not seen at concentration of 10, 30, 300, and 500 μM. This suggested that there is a difference in affinity as a blocker of the Nav1.5 VGSC within a concentration range of 50–100 μM^1^.

In that same study, it has been demonstrated that citalopram and both of its enantiomers modulated the activation and inactivation states of the Nav1.5 VGSCs, and after comparing the results along with their IC50, escitalopram has proved to contribute more than R-citalopram to the inhibitory effect on the channel current caused by citalopram^1^.

Nonetheless, this inhibitory effect on the Nav1.5 peak current might synergize with the inhibition of hERG K+ channels and potentiate the prolongation of the QTc-interval, increasing the risk of development of fatal arrhythmias like TdP.

On the other hand, Cav1.2, L-type calcium channels, are crucial components of cardiovascular function. They dominate the functional activity of the working myocardium by maintaining the action potential plateau, accelerating pacemaker activity in the SA node, and supporting conduction through the AV node. Because of their significance in normal cardiac and cardiovascular function, testing drug effects on their activity is critical to screen for unwanted cardiac side effects^7^.

It has been shown that Cav1.2 channels hyperactivity brings about myocardial hypertrophy, which leads to heart failure and hypertension. In addition, Cav1.2 gene mutations can lead to disease occurrence manifested by the prolongation of the QT-interval^7^. Voltage-gated, L-type CaV1.2 channels play a critical role in cardiac excitation-contraction (EC) coupling by dictating the degree of Ca2+ influx into cardiomyocytes, which in turn determines the magnitude of ventricular contraction. The trafficking and surface expression of CaV1.2 channels are tightly regulated through various pathways, including biosynthetic delivery, endosomal recycling, ubiquitination, and degradation^11^.

Recent research suggests that alterations in CaV1.2 trafficking dynamics could be linked to cardiotoxicity^11^. For instance, prolonged activation of angiotensin II type 1 receptors (AT1R) can lead to the endocytosis of CaV1.2 channels, reducing their surface expression and thereby decreasing ICa. This process involves *β*-arrestin1 recruitment and subsequent internalization, which could potentially protect against Ca2+ overload and associated cardiac dysfunction.

Numerous other mechanisms have been implicated in the cardiotoxicity associated with citalopram and escitalopram. Notably, certain metabolites of these drugs, including desmethylcitalopram and ultimately didesmethylcitalopram^12^, have been identified in the human body. Didesmethylcitalopram emerges as the primary metabolite responsible for the cardiac toxic effects^13^, manifesting in various cardiac abnormalities and ECG alterations. These include atrioventricular dissociation, junctional escape cardiac rhythms with sinus arrest, and widening of the QRS complex^14,15^.

However, recent studies have revealed that it is the parent compounds, not their demethylcitalopram and didemethylcitalopram metabolites, that elevate the risk of arrhythmias at therapeutic concentrations^16–18^. A cohort study conducted by Faraj et al. in 2023, involving 19,742 participants, demonstrated this phenomenon^16^. The research highlighted that impaired drug metabolism poses a greater risk than increased demethylcitalopram concentration, a trend particularly pronounced in individuals with the CYP2C19 phenotype, impacting citalopram and escitalopram serum concentrations.

### Citalopram and escitalopram toxicity: Predisposition

A known complication of treatment with many drugs, including citalopram and escitalopram, is QTc prolongation with increased risk of TdP and cardiac arrest. However, this is also noted in conditions of hypothermia^9^. Consequently, since these side effects do not occur in all patients, it is important to detect patients at risk of developing ventricular arrhythmias (tachycardia, fibrillation, TdP), and immediately act to reduce the risk of undesirable effects.

**Figure 2.**
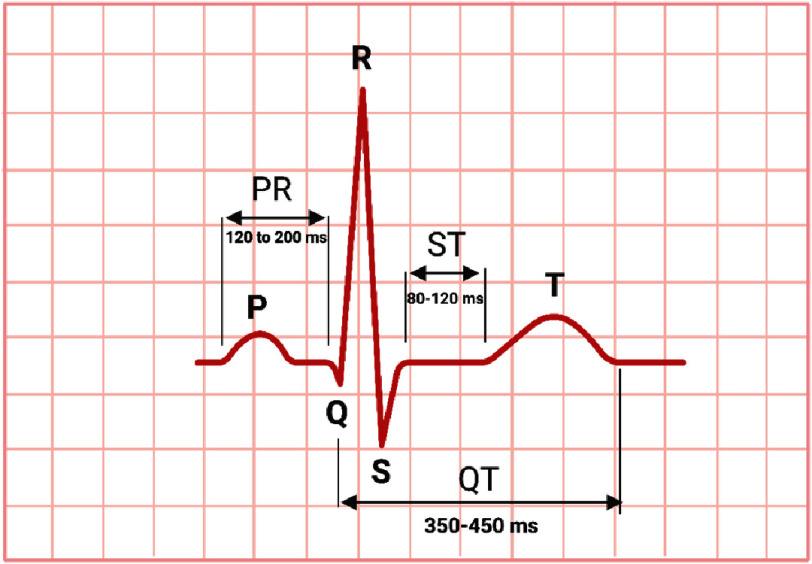
Normal ECG measures.

A novel ECG biomarker QRS/QTc is potentially able to predict the predisposition to such arrhythmias. It has been found that QRS/QTc <0.2 is associated with higher risk of hypothermic cardiac arrest which has similar electrophysiology to that of citalopram and escitalopram prolonging repolarization^9^. The QRS/QTc values would be low in patients taking high citalopram doses, above the threshold of hERG-inhibition and below VGSC channel inhibition. These patients are theoretically at higher risk of developing TdP as opposed to higher QRS/QTc values in patients with comorbidities like prolonged QRS (reduced excitability/conduction velocity), and prolonged QTc (delayed repolarization) (Figure 2)^9^.

Citalopram and escitalopram are metabolized by the hepatic enzyme CYP2C19. Normally, drug byproducts like demethylcitalopram and didemethylcitalopram circulate in the plasma at low amounts and do not contribute to the pharmacologic activity of the drug. However, patients with CYP2C19-mutations have higher serum ratio of the metabolites to the mother compound.

In dogs, didemethylcitalopram, associated with unexpected and sudden death, appeared to be the most cardiotoxic. Genetic variations along with drug interactions can therefore cause individual differences in cardiac toxicity by altering serum concentration of citalopram metabolites^9^. Since drug monitoring would not detect patients at risk unless metabolites concentrations were also monitored, QRS/QTc values could identify patients with CYP2C19 metabolism that predisposes patients towards pro-arrhythmic state when taking citalopram and escitalopram^9^. To conclude, and as we can denote from the following table, calculating QRS/QTc in the gathered cases proved that this emerging ECG-marker is capable of unveiling patients at risk of developing ventricular arrhythmias.

Ji et al. conducted a comprehensive examination of the CYP phenotypes through a genome-wide association study^19^. The study revealed genome-wide significant associations between escitalopram concentration and single nucleotide polymorphisms located in or near the CYP2C19 gene on chromosome 10 ( rs1074145, *P* = 4. 1 × 10 −9). Additionally, S-didesmethylcitalopram concentration showed significant associations with single nucleotide polymorphisms in proximity to the CYP2D6 locus on chromosome 22 ( rs1065852, *P* = 2. 0 × 10 −17). These findings underscore the pivotal role of CYP450 enzymes, specifically CYP2C19 and CYP2D6, in the biotransformation of citalopram. Ji et al. concluded that both *in vitro* and *in vivo* studies consistently suggest that the biotransformation of citalopram to monodesmethylcitalopram and didesmethylcitalopram is catalyzed by these CYP isozymes.

### Citalopram & escitalopram usage in geriatric population

The use of citalopram and escitalopram in the geriatric population presents somewhat contradictory evidence regarding their safety. While these medications are commonly prescribed for depression and anxiety in older adults, there is conflicting data about their association with QTc prolongation and related cardiac risks. Some studies suggest an increased risk of QTc prolongation and Torsades de Pointes (TdP) in the elderly, however other studies have not found any significant association between these drugs and adverse cardiac events in this population, which complicates the assessment of their safety profile. Here we discuss these conflicting findings and their implications to clarify the risks and guide clinical practice.

The use of citalopram and escitalopram has been associated in many observational studies with an increased risk of QT-prolongation, which has led to warnings from regulatory agencies and setting of upper dosage limits by the FDA^4,10^. The risk of QT-prolongation and TdP has been long-known. It first appeared in syncope patients receiving quinidine, then it was reported with non-cardiac treatment drugs leading to withdrawing some drugs like terfenadine, astemizole, and cisapride from the market^20^. After many reports, the FDA worked on testing QT-prolongation potential, but that turned out to be unreliable because the absence of QT-prolongation on physiological measurement is not a warranty for safety^20^. Consequently, considering that accurately measuring a drug’s risk of QT-prolongation is impossible, the only safe reliable factor for administration of a drug is relying on a history of safe administration in large numbers of patients^20^.

In a 2019 study in a university-affiliated geriatric health care center, electronic health records, EHR, of patients over a 7-year period (April 2008 to July 2015) were reviewed and patients on citalopram and escitalopram with ECG within 24 h to 90 days or patients already on the medication but had a dose increase or decrease were eligible – excluding patients with diagnosed atrial fibrillation^10^. Of 3,809 patients taking antidepressants during the study period, 1,065 were prescribed citalopram and escitalopram, and 137 met the study criteria: with 97 on citalopram and 40 on escitalopram. Complications were mainly divided into TdP and sudden cardiac death, SCD.

In this review, there were no cases of TdP. On the other hand, only one patient taking 15mg escitalopram followed by risperidone (antipsychotic medication) had SCD^10^. In conclusion, in this study, no association could be found between citalopram, escitalopram and QTc. So, although the literature reveals evidence of an association between citalopram and QTc prolongation in the general adult population, this association has still not been detected in the geriatric population^10^.

Another observational study was carried out on a 20% random sample, 1,265,921 patients (65-year-old and above) comprised of Medicare beneficiaries from 2007–2016^20^. In this study, drugs known to have a risk of TdP like fluoroquinolones, macrolides, SSRIs, and SNRIs were followed among these patients to monitor for adverse heart events. Patients were followed until recording ventricular arrhythmia (VA), SCD, death, disenrollment from Medicare, switch to capitated plan or year end 2016^20^.

**Figure 3.**
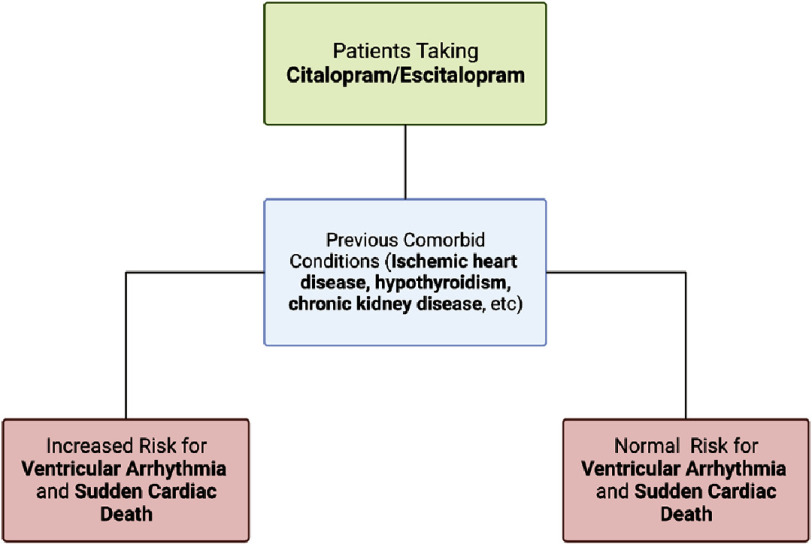
Predisposition to cardiac toxicity in patients taking citalopram/escitalopram.

Ischemic heart disease (28.3%), hypothyroidism (20.7%), and chronic kidney disease (19.1%) were found to be the most associated proarrhythmic comorbidities (Figure 3)^20^. Although both citalopram and escitalopram users had an increased risk for VA and SCD, escitalopram was associated with a higher risk: 31% increased risk compared to former users, 21% compared to SNRIs users, and 18% compared to never-users^20^.

To note, the risk of cardiac adverse effects was decreased in patients with a cumulative use of 12 months or more. For example, in patients with less than 12-month cumulative usage, the risk is increased by 23% compared to a 14% risk reduction in patients with more than 12 months cumulative usage^20^. On the other hand, escitalopram had a 34% increased risk compared to non-users, while the risk decreased to 4% in patients with more than 12 months usage^20^. By comparing citalopram and escitalopram, escitalopram has shown to be associated with a higher risk than citalopram: citalopram is safer with a 13% reduction of risk^20^.

In a 2022 study on data from the Danish national healthcare registries (January 2002 to December 2016) including patients aged 65 and above, patients were followed from the prescription until the occurrence of serious arrhythmias^4^. A total of 225 events of serious arrhythmias were recorded: 147 in citalopram initiators, 32 in escitalopram and 46 in the comparator group (initiators of other SSRIs). However, there was no increased risk of adverse cardiac events among citalopram and escitalopram initiators compared to other SSRIs^4^. Conversely, in an older population-based study from 2016, it has been shown that the initiation of citalopram is associated with a small but statistically significant increased risk of hospital encounter with ventricular arrhythmia after 90 days, as compared to other prescribed SSRIs, which might have increased the risk of 90-days death^21^.

### Relationship between QTc-prolongation and hemodialysis

Although SSRIs are known for their potential to prolong the QT-interval, they are mostly used by the hemodialysis population^22^. On the other hand, more than 35% of hemodialysis patients are given proton pumps inhibitors (PPIs) which, in turn, interact with citalopram and escitalopram both pharmacodynamically, altering pharmacologic effect, and pharmacokinetically, being competitive inhibitors of the CYP 2C19 responsible for their metabolism^5^. In addition, PPIs can cause hypomagnesemia, which as an electrolyte abnormality, causes QT-prolongation^5^.

A retrospective cohort study from the U.S Renal Data System (USRDS) looked for QT-prolongation in patients on hemodialysis taking citalopram and escitalopram compared to sertraline (as a control) since it is not metabolized by the CYP 2C19 system. A total of 72,559 patients on maintenance hemodialysis was included. Among these, 14,983 (21%) are citalopram/escitalopram users using PPI, 26,503 (36%) citalopram/escitalopram users not using a PPI, 10,779 (15%) sertraline users using a PPI, and 20,294 (28%) sertraline users not using a PPI. Relative to sertraline initiation without PPI, initiation of citalopram/escitalopram with PPI use has been linked with the highest risk of SCD (death from arrhythmia), followed by citalopram administration without PPI. Conversely, sertraline initiation with PPI use was not associated with SCD^5^. To conclude, PPI use in hemodialysis patients on citalopram/escitalopram may potentiate the risk for arrhythmia and SCD which suggests drug-drug interaction, which requires further research in that field^5^.

Without drug interactions, SCD is 20–30 times more likely to occur in hemodialysis patients compared to the general population, mainly due to hypokalemia which causes arrhythmia^22^. Available data showed that citalopram/escitalopram are associated with a higher risk of SCD compared to other SSRIs with lesser QT-prolongation risk^22^.

Hemodialysis patients have larger serum-to-dialysate potassium gradients, which in the setting of QT-prolonging drugs like citalopram/escitalopram, may increase their proarrhythmic potential and SCD risk^21^. A comparative study using data from USRDS compared the risk of SCD among patients exposed or not exposed to larger baseline serum-to-dialysate potassium gradients taking citalopram/escitalopram *versus* other patients taking other SSRIs with lower proarrhythmic potential like fluvoxamine, sertraline, fluoxetine, and paroxetine^22^. 25,099 patients on hemodialysis were included: 11,107 (44.3%) high-QT prolongation potential SSRI and 13,992 (55.7%) lower QT-prolongation potential SSRI users. As a result, patients on high QT-prolongation risk SSRIs with higher serum-to-dialysate (>4 mEq/l) had 443 SCDs reported in the 1-year follow-up period, *versus* 186 SCD events in the patients on citalopram/escitalopram and a lower baseline gradient (<4 mEq/l). On the other hand, 15 SCD events were reported in lower QT-prolongation risk SSRIs with baseline >4 mEq *versus* 216 events with a baseline <4 mEq/l^22^. In conclusion, the potential for QT-prolongation in SSRIs is modified by the serum-to-dialysate potassium gradient, where higher citalopram/escitalopram had more than double the risk for QT-prolongation in a higher baseline (>4 mEq/l) as compared to lower-risk QT-prolongation SSRIs^22^.

In contrast, in a lower baseline (<4 mEq/l) setting, SCD risk was similar among citalopram/escitalopram and lower QT-prolongation potential SSRIs^22^. These findings suggest that minimizing the serum-to-dialysate potassium gradient in the setting of citalopram/escitalopram administration can be warranted, but further studies are needed to investigate for the effect of serum-to-dialysate potassium gradients in the setting of administration of other drugs with QT-prolongation potential.

## Conclusions

This review has highlighted that citalopram and escitalopram have potential for cardiotoxicity by interfering with essential cardiac ion channels. Identifying at-risk individuals through factors such as the QRS/QTc ratio and genetic variations in CYP2C19 metabolism is pivotal for pre-emptive measures against cardiac complications. It is noteworthy that the relationship between QTc prolongation and these medications may not exhibit consistency among geriatric patients, necessitating further investigation. Furthermore, in the context of hemodialysis, the interaction with proton pump inhibitors and the serum-to-dialysate potassium gradients compound the risk of arrhythmias in hemodialysis patients on citalopram/escitalopram, underscoring the imperative of precise management to mitigate adverse outcomes. In essence, while citalopram and escitalopram remain valuable antidepressants, their potential for cardiotoxicity, particularly within specific populations, underscores the importance of vigilant monitoring and ongoing research to enhance safety within clinical practice.

## Acknowledgements

**Conceptualization:**  Hadi Farhat, Lea Nassif, Sai Dheeraj Gutlapalli, Razan Abdulaal

**Data curation:**  Yehya Tlaiss

**Formal analysis:**  Hadi Farhat, Lea Nassif, Yehya Tlaiss

**Investigation:**  Hadi Farhat, Lea Nassif, and Sai Dheeraj Gutlapalli

**Methodology:**  Yehya Tlaiss.

**Project administration:**  Razan Abdulaal.

**Supervision:**  Razan Abdulaal

**Validation:**  Razan Abdulaal, Nour Bachaalany

**Visualization:**  Razan Abdulaal

**Writing - original draft:**  Hadi Farhat, Lea Nassif, Yehya Tlaiss

**Writing - review & editing:**  Yehya Tlaiss, Razan Abdulaal, Sai Dheeraj Gutlapalli, Nour Bachaalany

The authors wish to acknowledge the contribution of Nour Bachaalany to the revision of this manuscript.
